# Mapping research trends of insulin resistance in polycystic ovary syndrome from 2017 to 2021: A bibliometric analysis

**DOI:** 10.3389/fendo.2022.963213

**Published:** 2022-12-15

**Authors:** Yong Chen, Qian Zhang, Jinhui Ma, Yuexin Yu

**Affiliations:** Center of Reproductive Medicine, General Hospital of Northern Theater Command, Shenyang, China

**Keywords:** polycystic ovary syndrome, insulin resistance, bibliometrics, visual analysis, VOSviewer

## Abstract

**Introduction:**

To map publication trends and explore research hotspots of insulin resistance (IR) in polycystic ovary syndrome (PCOS) study.

**Methods:**

With the theme of “Polycystic ovary syndrome” AND “Insulin Resistance”, the key data set of Science Core Literature Collection (WoSCC) web from 2017 to 2021 was extracted and bibliometric analysis was performed. Through VOSviewer v1.6.10 software, the research trend in this field is analyzed visually.

**Results:**

2080 literatures about IR in PCOS from 2017 to 2021 were downloaded. The following basic information was collected for each article: country, author, institution, journal, references. The key words are divided into six categories: (1) The interaction between insulin resistance and chronic inflammation; (2) The relationship between insulin resistance and metabolic syndrome and nonalcoholic fatty liver disease; (3) The interaction between insulin resistance and hyperandrogenemia; (4) The relationship between insulin resistance and dyslipidemia; (5) Metformin may regulate insulin resistance in the treatment of PCOS; (6) The study of serum biomarkers in PCOS patients with insulin resistance.

**Discussion:**

The six key words extracted can provide an in-depth perspective for the study of IR in PCOS, and provide valuable information to help researchers identify potential research directions, collaborators and cooperative institutions.

## Introduction

Polycystic ovary syndrome (PCOS) is a common reproductive endocrine disease in women, affecting 5% to 20% of women of childbearing age (1, 2). PCOS is one of the most complex and difficult diseases in the field of gynecologic endocrinology, which is characterized by uncertain and complex etiology, highly heterogeneous clinical manifestations, and nonspecific clinical treatment methods (3). Insulin resistance (IR), as the main metabolic characteristic of PCOS, is considered an important pathophysiological basis involved in the pathogenesis of PCOS. Many studies had focused on possible relationships between IR and PCOS (4). Available data on the molecular defects of IR, such as extracellular signal-regulated kinase (ERK) and AMP-activated protein kinase (AMPK) signal pathways involved in IR are inconsistent in PCOS women, suggesting heterogeneity mechanisms of IR involve in PCOS (5–7). The lack of accurate measures for IR and the heterogeneity of PCOS disease has blurred the relationship between IR and PCOS (8). Several family studies show that PCOS typical endocrine and metabolic characteristics are frequent in PCOS women relatives, which refer to the genetic and epigenetic mechanisms of PCOS (9). Putting all the puzzle together, it is unable for us to conclude the definitive association between IR and PCOS.

Furthermore, IR and hyperandrogenism, abnormal lipid metabolism, oxidative stress, nonalcoholic fatty liver disease (NAFLD) are intricate interactions that further promote the development and development of PCOS. Defining the molecular mechanism of IR may provide new perspectives and strategies for the treatment of PCOS.

In recent decades, numerous research papers related to IR in PCOS have been published in academic journals. This study uses bibliometric methods and mapping knowledge domains (MKD) methods to explore the current status of IR-related research in PCOS. Bibliometric analysis is a method of analyzing relevant documents using mathematical methods. It can make statistical data on the distribution, correlation and clustering of relevant documents to quantitatively measure relevant documents (10). Using database and visualization technology, the MKD method provides a new way for literature mining and revealing the core structure of scientific knowledge. In recent years, co-citation analysis and keyword co-occurrence analysis have been used in knowledge analysis maps. Thus, this study assesses the growth of publications, international collaborations, authors, journals, citations, and keyword co-occurrence analyses relevant to IR research in PCOS. Evaluating research trends in an academic field is an important element for researchers to explore. Bibliometric hotspot analysis can be used as an intuitive tool to assess important trends in research and identify importance. Therefore, the aim of this study is to make a comprehensive analysis of the scientific literature related to IR in PCOS.

## Materials and methods

### Data source and research process

The Science Citation Index Extended Database was searched on the Science Core Literature Collection (WoSCC) web as a source of research. The search keywords were “Polycystic ovary syndrome” AND “Insulin resistance”, the document type was “journal article” which including all kinds of literature. And the time span was “Jan 1 2017 to Dec 31 2021”. No language and species limit is set. The retrieved results are saved as a “txt” file containing “Full Records and References”. The following basic information was collected for each article: country, author, institution, journal, references and keywords.

### Analytical tool and method

In this study, the above-mentioned downloaded data were imported into VOSviewer v.1.6.10 for systematic analysis. VOSviewer (http://www.vosviewer.com) is a document visualization software developed by Van Eck and Waltman in 2010, which has the advantage of showing the results of cluster analysis (11–14). In the knowledge map generated by the VOSviewer, research projects are presented as nodes and links such as country, organization, author, co-cited reference and keywords, etc. The relationships between research projects can be demonstrated through nodes and links. In the present study, bibliographic co-citation analysis and keyword co-occurrence analysis networks were used to construct a knowledge map of IR studies in PCOS. Reference co-citation cluster analysis can be used to summarize the main topics in this research area. In the keyword co-occurrence analysis, keywords can express the theme of the literature, and the cluster analysis of these co-occurrence keywords can reveal the knowledge structure and research hotspots in this field.

## Results

### Annual distribution of publications

**Figure 1:**
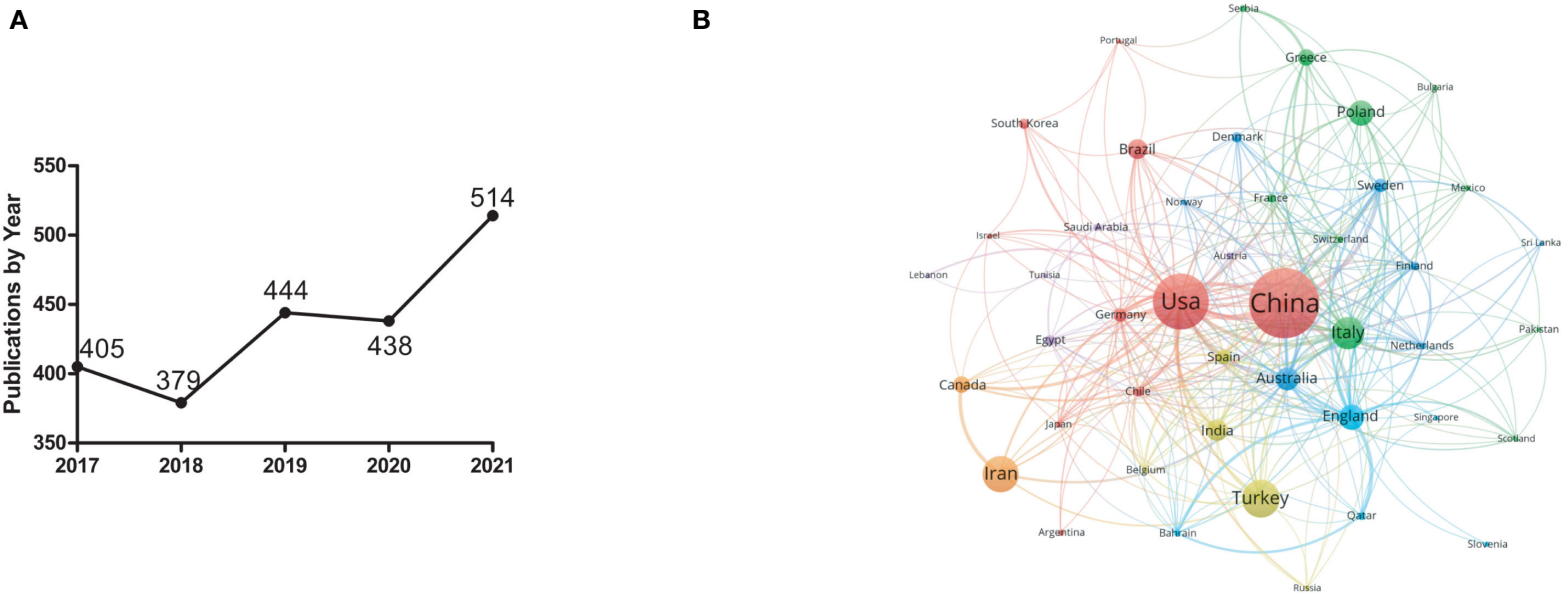
General characteristics of IR studies in PCOS from 2017 to 2021. **(A)** Annual publications on IR studies in PCOS. **(B)** Main country distribution of IR studies in PCOS. The minimum threshold for the number of publications was set to 5. Of the 84 countries participating in IR studies in PCOS, 46 countries have reached the minimum number of publications.

According to the bibliometric search results, 2080 articles related to IR research in PCOS were collected from Jan 1 2017 to Dec 31 2021. Over the past five years, the number of papers published has generally leveled off, with the highest number of papers published in 2021 at 482 (**Figure 1A**).

### Country analysis

According to the search results, 2080 articles were from 84 countries. As shown in **Table 1**, the top 10 countries engaged in IR research in PCOS published 1638 articles, accounting for 86.3% of the total number of publications. China had the largest number of articles published with 425,22.4%), followed by the United States (309,16.3%) and Turkey (178,9.4%). According to the citation analysis, there were 4010 citations in the United States, followed by China (2961 citations) and Italy (2038 citations).

**Table 1:** Top 10 Countries with IR Research-Related Articles Published in PCOS from 2017 to 2021.

Rank	Country	Count(%)	Citation
1	China	425(22.4)	2961
2	USA	309(16.3)	4010
3	Turkey	178(9.4)	1188
4	Iran	167(8.8)	1254
5	Italy	142(7.5)	2038
6	Poland	102(5.4)	544
7	England	99(5.2)	1324
8	Australia	87(4.6)	1402
9	India	81(4.3)	566
10	Spain	48(2.5)	569

The country co-authorship analysis reflects the degree of communication between countries and between influential countries in the field. The larger the node, the greater the influence of the countries represented in this field; the thickness and distance of the connections between nodes represent the cooperative relationship between countries. As shown in **Figure 1B**, China has developed intensive cooperation with the United States, Italy, Australia, Switzerland and other countries in the field of IR research in PCOS.

### Distribution of major research institutions

**Figure 2:**
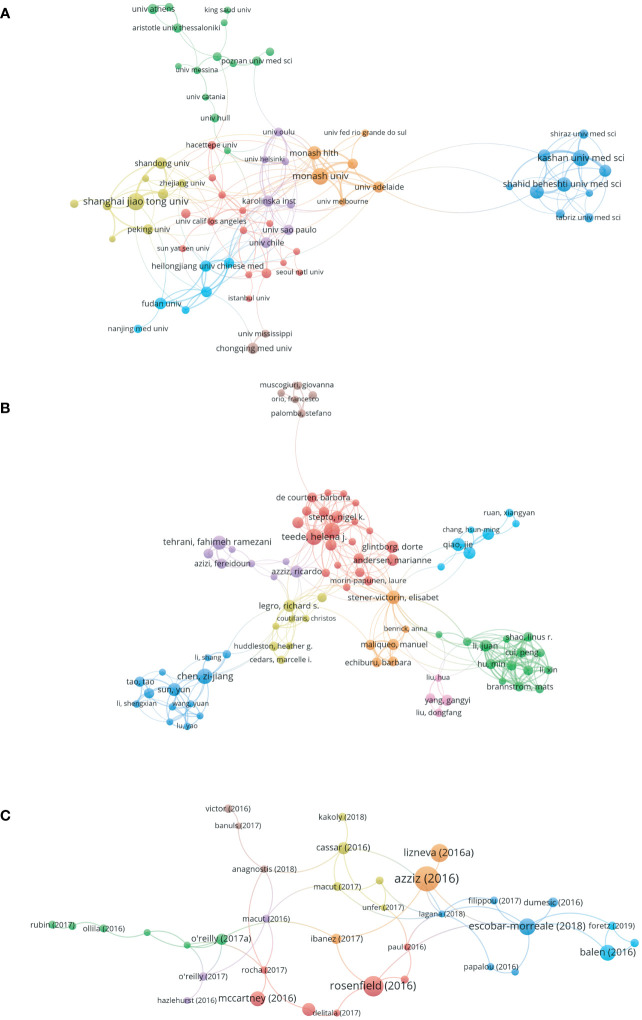
Co-occurrence authors and citations of IR studies in PCOS from 2017 to 2021. **(A)** Collaboration network of the main research institutions in the IR-related literature in PCOS. The minimum threshold for the number of publications was set at 10. Of the 2067 organizations participating in the study, 77 reached the threshold. **(B)** Network of authors of IR-related studies in PCOS. The minimum threshold for the number of publications per author was set at 10. Of the 8266 authors involved in IR-related studies in PCOS, 43 reached the minimum threshold. **(C)** Literature citation analysis of IR-related studies in PCOS. The minimum number of references threshold is set to 30. Of the 2080 articles, 77 reached the threshold.

According to the search results, 2080 articles were published by 2067 organizations. Among them, the top ten organizations published 345 articles, accounting for 18.2% of the total (**Table 2**). Based on the co-occurrence analysis of research institutions, **Figure 2A** shows a knowledge domain map of the distribution of research institutions for IR-related literature in PCOS. The size of the node corresponds to the number of published articles. Links between nodes represent collaboration. The stronger the link, the closer the collaboration.

**Table 2:** Top 10 Research Institutions in PCOS from 2017 to 2021.

Rank	Research Institutions	Country	Count(%)	Citation
1	Shanghai Jiaotong University	China	50(2.6)	374
2	Monash University	Australia	49(2.6)	881
3	Kashan University	Iran	45(2.4)	500
4	Tehran University of Medical Science	Iran	36(1.9)	274
5	Monash Health	Australia	33(1.7)	686
6	Iran University of Medical Sciences	Iran	29(1.5)	371
7	Arak University of Medical Sciences	Iran	28(1.5)	321
8	Shangdong University	China	26(1.4)	427
9	University of Adelaide	Australia	25(1.3)	419
10	Karolinska Institutet	Sweden	24(1.3)	324

### Analysis of the main authors of the literature

Among all authors, Asemi Z published 41 articles, ranking first. This was followed by Jamiliam M (21 articles) and Teede H (21 articles), showing their fruitful contribution to the study of IR in PCOS. It is worth noting that the Chinese authors, Professor Chen Zijiang and Professor Qiao Jie, are ranked fifth (19 articles) and tenth (14 articles). In addition, the authors’ co-cited information was analyzed. Among all cited authors, Azziz R was cited 910 times, ranking first, followed by Legro RS (860 co-citations) and Diamanti KE (808 co-citations), indicating their impact in the area of IR study in PCOS (**Table 3**). Based on the co-authorship analysis, **Figure 2B** shows a knowledge domain map of the distribution of authors in the area of IR study in PCOS. The size of the node corresponds to the number of published articles. The connection between the nodes represents a cooperative relationship between the authors. The greater the link strength, the higher the cooperation density.

**Table 3:** Top 10 Authors and Co-cited Authors of IR Research Related Articles Published in PCOS from 2007 to 2021.

Rank	Author	Count(%)	Co-cited Author	Count(%)
1	Asemi, Z	41(2.2)	Azziz, R	910
2	Jamilian, M	21(1.1)	Legro, RS	860
3	Teede, H	21(1.1)	Diamanti-kandarakis, E	808
4	Moran, L	20(1.1)	Fauser, B	682
5	Chen ZJ	19(1.0)	Moran, L	568
6	Atkin, S	19(1.0)	Dunaif, A	559
7	Sathyapalan, T	18(0.9)	Chang, J	467
8	Tehrani, F	16(0.8)	Escobar-morreale, H	442
9	Escobar-morreale, H	15(0.8)	Ehrmann, D	396
10	Qiao J	14(0.7)	Gonzalez, F	396

### Analysis of major source journals

Based on the search results, articles on IR-related research in PCOS were published in 505 journals. **Table 4** lists the journals with the top 10 publications on this topic. Gynecological Endocrinology published the most articles (146,7.7%), followed by Clinical Endocrinology (49,2.6%) and Fertility Sterility (46,2.5%). Articles published in these three journals accounted for 12.8% of all publications in this study.

**Table 4:** Top 10 Journals of IR Research Related Articles Published in PCOS from 2007 to 2021.

Rank	Journal	Country	Count(%)
1	Gynecological Endocrinology	England	146(7.7)
2	Clinical Endocrinology	England	49(2.6)
3	Fertility and Sterility	USA	46(2.4)
4	Journal of Clinical Endocrinology and Metabolism	USA	43(2.3)
5	Frontiers in Endocrinology	USA	36(1.9)
6	Journal of Endocrinological Investigation	Italy	36(1.9)
7	Hormone and Metabolic Research	Germany	31(1.6)
8	Endocrine Connections	USA	29(1.5)
9	Journal of Obstetrics and Gynaecokogy	England	28(1.5)
10	Human Reproduction	England	26(1.4)

### Citation distribution

Through the co-citation analysis of the cited documents of IR-related research in PCOS, the research foundation in this field can be effectively constructed. Of the 52963 cited references, 366 citations reached cutoff values. The top 10 references are shown in **Table 5**. The minimum number of citations for a single document was set at 30; by analyzing the citation frequency of 2080 documents, 77 documents reached the threshold (**Figure 2C**). The size of the node corresponds to the frequency of references.

**Table 5:** The top 10 citations in the literature on IR-related studies in PCOS from 2017 to 2021.

Rank	Title	Citations
1	Revised 2003 Consensus on Diagnostic Criteria and Long-Term Health Risks Related to PCOS.	467
2	Revised 2003 (2004) Consensus on diagnostic criteria and long-term health risks related to polycystic ovary syndrome (PCOS).	429
3	Insulin Resistance and the Polycystic Ovary Syndrome Revisited: An Update on Mechanisms and Implications.	273
4	Homeostasis model assessment: insulin Resistance and Beta-Cell Function from Fasting Plasma Glucose and Insulin Concentrations in Man.	271
5	Diagnosis and Treatment of Polycystic Ovary. Syndrome: An Endocrine Society Clinical Practice Guideline.	218
6	The Prevalence and Features of the Polycystic Ovary Syndrome in Unselected Population.	210
7	The Prevalence of Polycystic Ovary Syndrome in a Community Sample Assessed under Contrasting Diagnostic Criteria.	207
8	Serum Unconjugated Bisphenol A Concentrations in Women May Adversely Influence Oocyte Quality during in Vitro Fertilization.	188
9	The Androgen Excess and PCOS Society Criteria for the Polycystic Ovary Syndrome: The Complete Task Force Report.	185
10	Impaired glucose tolerance, type 2 diabetes and metabolic syndrome in polycystic ovary syndrome: a systematic review and meta-analysis.	178

### Distribution of keywords: Hotspots of IR in PCOS study

**Figure 3:**
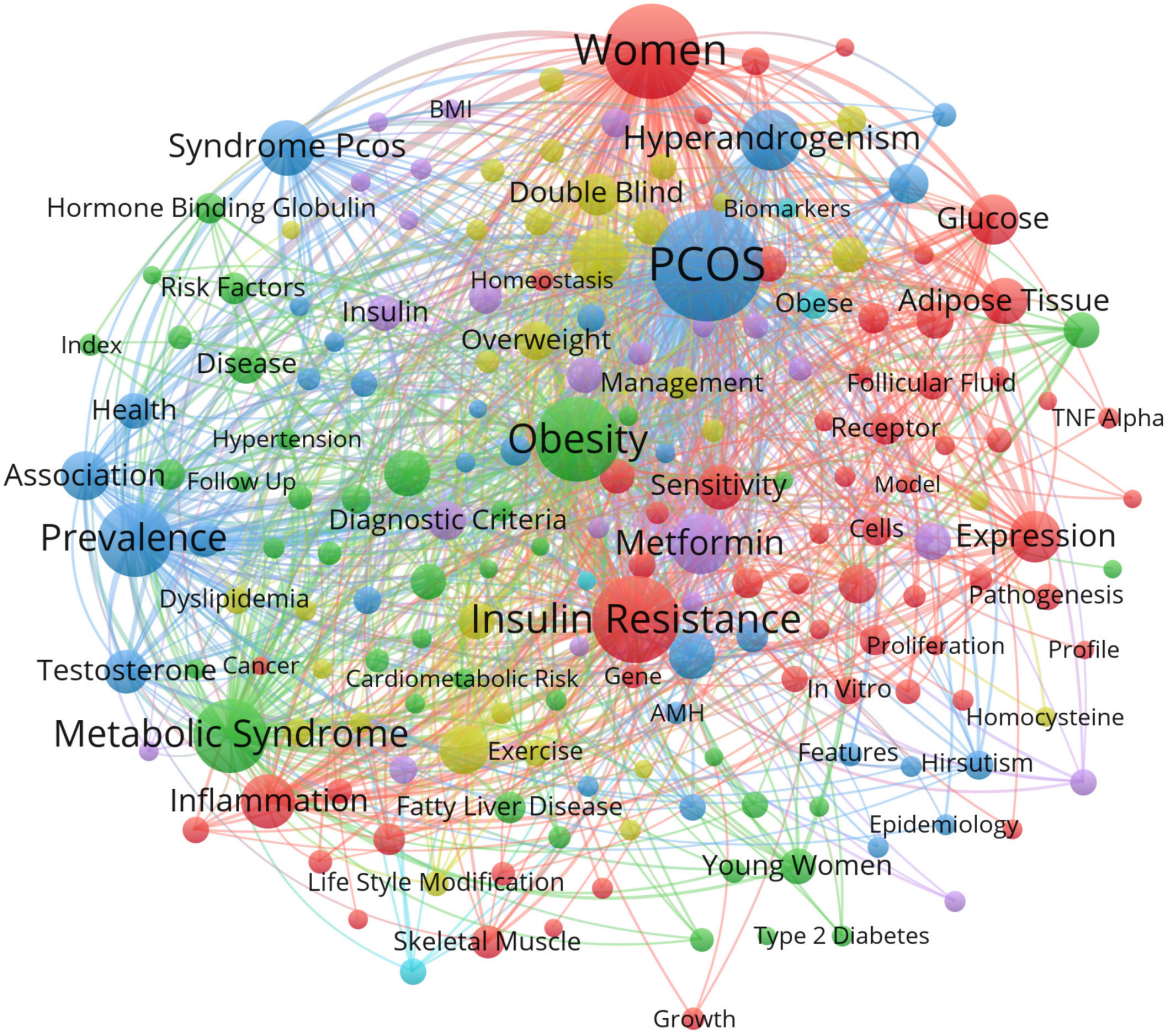
Key-word co-occurrence network for IR-related studies in PCOS from 2017 to 2021. The minimum number of keyword occurrences threshold is set to 15. Of the 5334 keywords involved in IR in PCOS, 217 reached the threshold.

Through high frequency keyword co-occurrence analysis, IR-related research hotspots in PCOS can be identified. The minimum co-occurrence threshold for keywords is set to 15. Of the 5334 keywords extracted for IR-related studies in PCOS, 217 reached the threshold. Base on this network, keywords with similarity were clustered and the six main clusters were represented in red, green, pink, blue, yellow and gray respectively (**Figure 3**). **Table 6** lists the top 10 keywords for each cluster.

**Table 6:** Co-occurence analysis of keywords. Top 10 keywords in the 6 clusters.

Cluster 1(red)	Cluster2(green)	Cluster 3(blue)	Cluster 4(yellow)	Cluster 5(pueple)	Cluster 6(sky blue)
Insulin Resistance (216)	Obesity (216)	PCOS(215)	Obese Women (186)	Metformin (205)	Obese(121)
Women(211)	Metabolic Syndrome (208)	Prevalence (203)	Oxidative Stress(175)	Diagnostic Criteria(164)	Plasma(94)
Inflammation(179)	Impaired Glucose Tolerance (163)	Hyperandrogenism(198)	Double Blind(152)	Insulin(144)	Biomarkers (60)
Glucose(174)	Disease (145)	Syndrome PCOS(193)	Overweight (144)	Pregnancy (140)	Body Composition(55)
Adipose Tissue(174)	Glucose Tolerance(134)	Association (173)	Insulin Sensitivity (140)	Infertility (128)	
Sensitivity (168)	Young Women (126)	Androgen Excess(170)	Weight Loss(135)	Therapy(119)	
Expression (164)	C Reactive Protein (121)	Testosterone (168)	Meta Analysis (133)	Diabetes (110)	
Metabolism (144)	Fatty Liver Disease (113)	Criteria(141)	Management (129)	Dchiroinositol (109)	
Resistance (133)	Risk Factors (113)	Health(131)	Serum(103)	Hyperinsulinemi-a(109)	
Gene Expression (131)	Population (104)	Consensus (129)	Dyslipidemia (103)	In Vitro Fertilization(83)	

The key words are divided into six categories:(1) the interaction between IR and chronic inflammation and adipocyte metabolic disorders;(2) the relationship between IR and metabolic syndrome and NAFLD;(3) the interaction between IR and hyperandrogenism;(4) the relationship between IR and abnormal lipid metabolism;(5) the therapeutic effect of metformin on PCOS by regulating IR;(6) Study of serum biomarkers in patients with IR in PCOS.

## Discussion

The change in the number of academic papers is an important research indicator reflecting the development trend in this field. As shown in **Figure 1A**, 2080 papers were searched for IR-related studies in PCOS from 2017 to 2021. The analysis of published countries in **Table 1** shows that China accounts for the 22.4% of the total number of publications and is the country with the largest number of publications. This indicates that China is an international scientific center for IR-related research in PCOS.

Through the analysis of the distribution of research institutions, it is possible to identify the most representative research institutions and intra-institutional cooperation relationships in specific field. As shown in **Figure 2A**, Shanghai Jiaotong University published the largest number of documents (50 articles), followed by Monash University (49 articles) and Kashan University (45 articles), indicating that these research institutions are the core of the entire IR study. In addition, the establishment of a network knowledge map of co-authors provides possible opportunities for researchers to collaborate. As shown in **Figure 2B**, the red group was centered on Professor Teede H. The green group is centered on Professor Cui Peng; the pink group is centered on Professor Yang Gangyi; the yellow group is centered on Professor Legro R; the purple group is centered on Professor Tehrani F; the blue group is centered on Professor Chen Zijiang; the orange group is centered on Professor Stener V and the skyblue group is centered on Professor Qiao Jie. The analysis of the distribution of academic journals allows the identification of core journals in specific field. Gynecological Endocrinology was the most published journal in this study.

The co-citation analysis of numerous cited references can effectively show the research background in this field. Therefore, we performed a cluster analysis of the citations of the 2080 articles downloaded to explore the main topics of IR-related research in PCOS. As shown in **Table 5**, the top 10 cited references include the diagnostic criteria for PCOS, the relevant mechanisms of IR in PCOS, and clinical trials. The effects of IR and insulin concentration on β-cell dysfunction and fasting blood glucose in PCOS patients were mainly introduced. Studies on PCOS-related clinical trials suggest that PCOS is highly correlated with impaired glucose tolerance, type 2 diabetes and metabolic syndrome. Notably, the article entitled “Women with polycystic ovary syndrome have intrinsic insulin resistance on euglycaemic- hyperinsulaemic clamp”. Although it is not part of the top 10 cited references, it ranks ninth in link weights, indicating the correlation between intrinsic IR and body mass index in PCOS.

Lifestyle effective treatment intervention in PCOS also plays an important role in the structure of the knowledge map.

The co-occurrence of keywords was considered to represent the search topic and reveal the internal structure and research frontier of related fields. As shown in **Figure 3**, IR-related studies in PCOS mainly form six clusters, and keywords with similarity in the research topics are clustered. Based on the characteristics and current status of IR-related studies in PCOS, six clusters were analyzed as follows:

The first category of keywords (red) mainly includes the interaction of IR with chronic inflammation and adipocyte metabolic disorders in PCOS. It has been shown that chronic inflammation promotes hyperinsulinemia through a variety of inflammatory factors such as IL-8 and C- reactive protein (15). In addition, adipocytes with metabolic dysfunction are expressed in PCOS patients (16). Adiponectin is an adipokine with insulin sensitivity and anti-inflammatory effects expressed by adipocytes (17). It also regulates follicular growth and ovarian hormone synthesis (18). Downregulation of adiponectin expression in adipocytes with metabolic dysfunction in PCOS is associated with increased sympathetic activity, chronic inflammation, and IR (19). Sepiliand Nagamani reported that serum adiponectin levels in PCOS patients were negatively correlated with IR. Yildiz Y et al. confirmed the relationship between IR and decreased adiponectin levels, suggesting that it may be a marker of IR and related metabolic disorders (20). Therefore, chronic inflammation and adipocyte metabolic dysfunction may act together on the formation of IR in PCOS.

The second category of keywords (green) mainly includes the relationship between IR and metabolic syndrome and NAFLD/nonalcoholic steatohepatitis (NASH) in PCOS. Previous studies have shown that hyperresponsiveness of islet beta cells to adverse environmental factors leads to hyperinsulinemia and is a major driver of metabolic syndrome. Metabolic syndrome-related diseases, NAFLD/NASH and PCOS are the combined effects of hyperinsulinemia and metabolic syndrome (21). Recent studies have shown that both PCOS and NAFLD are strongly associated with IR, metabolic syndrome, and obesity (22, 23). IR was observed in approximately 50% to 80% of patients with PCOS and up to 80% of patients with NAFLD (24, 25).

Intuitively, one would assume that IR is always associated with obesity, but clinical evidence suggests the presence of IR in both obese and non-obese PCOS (26, 27). Diverse dietary patterns can improve obesity and IR are beneficial to PCOS patients (28). Mediterranean diet protects against IR-related diseases, such as obesity, NAFLD, type 2 diabetes by improving insulin sensitivity and consider it as the optimal nonpharmacological strategies for PCOS treatment (29). Recent study showed low-GI diets can improve common clinical characteristic of PCOS including IR, acne, hirsutism and menstrual irregularities (30). Pulse-based diet has been widely used in clinical to improve insulin sensitivity and reduce IR (31).

IR is also a key feature linking NAFLD with metabolic syndrome, as mentioned earlier. In addition, NAFLD is considered a hepatic manifestation of metabolic syndrome (32), whereas PCOS is an ovarian manifestation of metabolic syndrome (23). PCOS and NAFLD share a common metabolic pathway that is affected by both obesity and IR.

The third category of keywords (blue) mainly includes the interaction between IR and hyperandrogenism in PCOS. The most prominent clinical manifestation of polycystic ovary syndrome is hyperandrogenism. Excessive androgens affect glomerular cell function and follicular development through complex mechanisms leading to obesity and IR. Most patients with PCOS with hyperandrogenism have a defect in steroid secretion, resulting in abnormal follicular development and failure in the selection of dominant follicles (33). The development of PCOS is caused by hyperandrogenism through different pathways. Hyperandrogenism causes a series of pathophysiological changes in PCOS, including IR, hyperinsulinemia, dyslipidemia and imbalance of LH/FSH ratio (34–36). These changes not only promote the development of PCOS alone, but also interact to form a vicious circle that induces PCOS. IR aggravates hyperandrogenism to promote PCOS. IR and hyperandrogenism in PCOS are usually associated with each other. Understanding the relationship between these two most salient features of PCOS can help uncover the pathogenesis of PCOS. Insulin is involved in promoting cell growth, proliferation and differentiation by mediating two signaling pathways: the phosphatidylinositol 3-kinase (PI-3K)/Akt pathway and the MAPK pathway (37). Insulin also promotes PCOS formation through PI-3K/Akt and MAPK signaling pathways. PI3K inhibition in follicular cells from patients with PCOS reduces 17 alpha-hydroxylase expression, suggesting that insulin may promote steroidogenesis *via* the PI3K pathway (38). The presence of specific, high-affinity insulin receptors in human theca suggests that insulin can directly mediate the physiological effects of theca cells. Previous studies have shown that the interaction between insulin and LH can up-regulate steroidogenic acute regulatory protein (STAR) and cytochrome P450 family 17 subfamily A member 1 (CYP17A1) mRNA expression, thereby increasing androgen levels (39). We found that IR decreased the expression of human villous trophoblast hormone binding protein (SHBG) and inhibited the mRNA expression of insulin receptor substrate 1 (IRS-1), insulin receptor substrate 2 (IRS-2), solute carrier family 2 member 4 (GLUT-4) and phosphoinositide-3-kinase regulatory subunit 1 (PI3Kp85α) (40). It is suggested that SHBG may be involved in systemic IR mediated by PI3K/Akt pathway. In addition, increased insulin reduces SHBG synthesis, thereby reducing its binding to testosterone, leading to hyperandrogenism (36). Thus, IR and hyperinsulinemia in patients with PCOS may lead to hyperandrogenism through multiple pathways.

The fourth category of keywords (yellow) mainly includes the relationship between IR and abnormal lipid metabolism in PCOS. Dyslipidemia is the most common metabolic abnormality in PCOS. The most common manifestation is atherosclerotic dyslipidemia typical of IR state, namely hypertriglyceridemia, decreased high density lipoprotein cholesterol level and elevated low density lipoprotein cholesterol (41). Previous studies have shown that insulin can synergize with human chorionic gonadotropin (HCG) to increase peptidyl-prolyl cis-trans isomerase (CYP17CYP17) and p450 levels, leading to hyperlipidemia (42). The three main sites of IR are muscle, liver and adipose tissue. IR begins in muscle tissue, accompanied by immune-mediated inflammatory changes and excessive free fatty acid production, causing ectopic lipid deposition (43, 44). With impaired muscle glucose uptake, excess glucose returns to the liver, increasing neonatal lipogenesis (DNL) and circulating free fatty acids, further promoting ectopic fat deposition and insulin resistance. Tissue IR in liver muscle leads to increased delivery of glucose substrates to the liver, triggering DNL, accompanied by associated inflammation and ectopic lipid deposition. IR in adipose tissue leads to increased lipolysis in adipocytes, leading to increased circulating FFA, further exacerbating steatosis and IR in muscle tissue (45).

The fifth category of keywords (purple) mainly includes the therapeutic effect of metformin on PCOS by regulating IR. Insulin sensitizers, especially metformin (MF), have been shown to be effective in the treatment of PCOS, improving reproductive dysfunction in such patients (46, 47). MF reduces hepatic gluconeogenesis by activating the AMPK pathway, which lowers blood glucose and enhances insulin sensitivity (48). Previous studies have found that metformin can improve the reproductive endocrine function of PCOS rats through AMPK α- sirtuin 1 (SIRT1) pathway, which may be the molecular mechanism of IR in PCOS and may become a therapeutic target for PCOS (49–51).

The sixth category of keywords (sky blue) included studies of serum biomarkers in patients with IR in PCOS. Diabetes mellitus, cardiovascular disease and metabolic disease syndrome IR are prevalent in patients with PCOS and are strongly associated with reproductive and metabolic complications of the syndrome. Some of the methods currently used to measure IR are very reliable but complex, such as hyperinsulinemic glucose clamp, while others are less precise but less traumatic and easy to implement, such as homeostasis model of assessment for insulin resistence index (HOMA-IR). Therefore, new markers are needed to assess IR more reliably. To date, studies have proposed a variety of biomarkers in serum to facilitate and improve the determination of insulin resistance. Many new molecules have been found to be closely related to PCOS pathophysiology and IR, such as adipocytokines (Adiponectin, Visfatin, Vaspin and Apelin), Copeptin, Irisin, serpin family E member 1 (PAI-1) and Zonulin. Many other proteins such as Resistin, Leptin, retinol binding protein 4 (RBP4), Kisspetin, and Ghrelin have been proposed as potential new biomarkers of IR in PCOS (52).

The strength of this study is to display emerging frontiers in IR research of PCOS. Based on VOSviewer’s map, we found that plenty topics have been studied earlier and are also in a leading position in research. These topics have become research hotspots in recent years and have the potential for further research. In particular, it may provide new perspectives and directions for the clinical diagnosis and management of PCOS with IR.

Even though our paper provides a comprehensive review of the publications on IR in PCOS from 2017 to 2021, similar to other bibliometric analyses, our paper has some limitations. First of all, our research only collected relevant articles from the Web of Science databases. Although these databases contain a wealth of literature resources, there may still be some publications have not been included. Second, there may be deviations in the author’s signature and the organization’s signature, resulting in a certain bias in the statistical results.

This study constructs a series of scientific maps of annual literature volume, country distribution, international collaboration, authors’ production, source journals, urban reference and keywords on IR-related research in PCOS. The key words extracted in this study can help researchers identify new topics and help them predict research directions. The results of this study may help researchers engaged in IR-related research in PCOS to select appropriate journal publications and co-authors or institutions.

## Author contributions

YY and YC conceived and designed the study. YC and QZ performed data acquisition and interpretation. YY, YC and JM wrote the paper. All authors contributed to the article and approved the submitted version.

## References

[1] Azziz R Carmina E Chen Z . Polycystic ovary syndrome. Nat Rev Dis Primersx (2016) 11(2):16057. doi: 10.1038/nrdp.2016.57 27510637

[2] Macut D Bjekić-Macut J Rahelić D Doknić M . Insulin and the polycystic ovary syndrome. Diabetes Res Clin Pract (2017) 130:163–70. doi: 10.1016/j.diabres.2017.06.011 28646699

[3] Moghetti P . Insulin resistance and polycystic ovary syndrome. Curr Pharm Des (2016) 22(36):5526–34. doi: 10.2174/1381612822666160720155855 27510482

[4] Moghetti P Tosi F . Insulin resistance and PCOS: chicken or egg? J Endocrinol Invest (2021) 44(2):233–44. doi: 10.1007/s40618-020-01351-0 32648001

[5] Højlund K Glintborg D Andersen NR Birk JB Treebak JT Frøsig C . Impaired insulin-stimulated phosphorylation of akt and AS160 in skeletal muscle of women with polycystic ovary syndrome is reversed by pioglitazone treatment. Diabetes (2008) 57:357–66. doi: 10.2337/db07-0706 17977950

[6] Hansen SL Svendsen PF Jeppesen JF Hoeg LD Andersen NR Kritensen JM . Molecular mechanisms in skeletal muscle underlying insulin resistance in women who are lean with polycystic ovary syndrome. J Clin Endocrinol Metab (2019) 104:1841–54. doi: 10.1210/jc.2018-01771 30544235

[7] Nelson-Degrave VL Wickenheisser JK Hendricks KL Asano T Fujishiro M, Legro RS . Alterations in mitogen-activated protein kinase and extracellular regulated kinase signaling in theca cells contribute to excessive androgen production in polycystic ovary syndrome. Mol Endocrinol (2005) 19:379–90. doi: 10.1210/me.2004-0178 15514033

[8] Dumesic DA Hoyos LR Chazenbalk GD Naik R Padmanabhan V Abbott DH . Mechanisms of intergenerational transmission of polycystic ovary syndrome. Reproduction (2020) 159:R1–R13. doi: 10.1530/REP-19-0197 31376813 PMC6989388

[9] Yilmaz B Vellanki P Ata B Yildiz BO . Diabetes mellitus and insulin resistance in mothers, fathers, sisters, and brothers of women with polycystic ovary syndrome: A systematic review and meta-analysis. Fertil Steril (2018) 110:523–33. doi: 10.1016/j.fertnstert.2018.04.024 29960703

[10] Zou X Yue WL Vu HL . Visualization and analysis of mapping knowledge domain of road safety studies. Accident Anal Prev (2020) 118:131–45. doi: 10.1016/j.aap.2018.06.010 29958121

[11] Van Eck NJ Waltman L . (2007). Advances in Data Analysis: Proceedings of the 30th Annual Conference of the German Classification Society, pp. 299–306. Springer: Freie Universitt Berlin, Germany.

[12] Perianes-Rodriguez A Waltman L Van Eck NJ . Constructing bibliometric networks: A comparison between full and fractional counting. J Informetrics (2016) 10(4):1178–95. doi: 10.1016/j.joi.2016.10.006

[13] Cao Z Zhang Y Luo JH Liao WQ Cheng X Zhan JH . A bibliometric analysis of publications on burn sepsis using VOSviewer. Front Med (Lausanne) (2022) :9. 971393. doi: 10.3389/fmed.2022.971393 36186821 PMC9515469

[14] Lin JP Ling F Huang P Chen M Song M Lu K . The development of GABAergic network in depression in recent 17 years: A visual analysis based on CiteSpace and VOSviewer. Front Psychiatry (2022) 13:874137. doi: 10.3389/fpsyt.2022.874137 35664493 PMC9157549

[15] Shorakae S Ranasinha S Abell S Lambert G Lambert E Courten B . Inter-related effects of insulin resistance, hyperandrogenism, sympathetic dysfunction and chronic inflammation in PCOS. Clin Endocrinol (Oxf) (2018) 89(5):628–33. doi: 10.1111/cen.13808 29992612

[16] Shorakae S Teede H de Courten B Lambert G Boyle J Moran LJ . The emerging role of chronic low-grade inflammation in the pathophysiology of polycystic ovary syndrome. Semin Reprod Med (2015) 33(4):257–69. doi: 10.1055/s-0035-1556568 26132930

[17] Raucci R Rusolo F Sharma A Colonna G Castello G Costantini S . Functional and structural features of adipokine family. Cytokine (2013) 61(1):1–14. doi: 10.1016/j.cyto.2012.08.036 23022179

[18] Comim FV Hardy K Franks S . Adiponectin and its receptors in the ovary: further evidence for a link between obesity and hyperandrogenism in polycystic ovary syndrome. PloS One (2013) 8(11):e80416. doi: 10.1371/journal.pone.0080416 24260388 PMC3832407

[19] Shorakae S Abell SK Hiam DS Lambert EA Eikelis N Jona E . High-molecular-weight adiponectin is inversely associated with sympathetic activity in polycystic ovary syndrome. Fertil Steril (2018) 109(3):532–9. doi: 10.1016/j.fertnstert.2017.11.020 29428305

[20] Yildiz Y Ozaksit G Serdar Unlu B Ozgu E Energin H Kaba M . Serum adiponectin level and clinical metabolic, and hormonal markers in patients with polycystic ovary syndrome. Int J Fertil Steril (2014) 7(4):331–6. PMC3901186 24520503

[21] Nolan CJ Prentki M . Insulin resistance and insulin hypersecretion in the metabolic syndrome and type 2 diabetes: Time for a conceptual framework shift. Diabetes Vasc Dis Res (2019) 16(2):118–27. doi: 10.1177/1479164119827611 30770030

[22] Baranova A Tran TP Afendy A Wang L Shamasaddini A Mehta R . Molecular signature of adipose tissue in patients with both non-alcoholic fatty liver disease (NAFLD) and polycystic ovarian syndrome (PCOS). J Transl Med (2013) 11:133. doi: 10.1186/1479-5876-11-133 23721173 PMC3681627

[23] Baranova A Tran TP Birerdinc A Younossi ZM . Systematic review: association of polycystic ovary syndrome with metabolic syndrome and non-alcoholic fatty liver disease. Aliment Pharmacol Ther (2011) 33:801–14. doi: 10.1111/j.1365-2036.2011.04579.x 21251033

[24] Legro RS Castracane VD Kauffman RP . Detecting insulin resistance in polycystic ovary syndrome: purposes and pitfalls. Obstet Gynecol Surv (2004) 59:141–54. doi: 10.1097/01.OGX.0000109523.25076.E2 14752302

[25] Ciba I Widhalm K . The association between non-alcoholic fatty liver disease and insulin resistance in 20 obese children and adolescents. Acta Paediatr (2007) 96:109–12. doi: 10.1111/j.1651-2227.2007.00031.x 17187615

[26] Stepto NK Cassar S Joham AE Hutchison SK Harrison CL Goldstein RF . Women with polycystic ovary syndrome have intrinsic insulin resistance on euglycaemic-hyperinsulaemic clamp. Hum Reprod (2013) 28:777–84. doi: 10.1093/humrep/des463 23315061

[27] Diamanti KE Dunaif A . Insulin resistance and the polycystic ovary syndrome revisited: an update on mechanisms and implications. Endocr Rev (2012) 33:981–1030. doi: 10.1210/er.2011-1034 23065822 PMC5393155

[28] Che X Chen Z Liu MQ Mo Z . Dietary interventions: A promising treatment for polycystic ovary syndrome. Ann Nutr Metab (2021) 77(6):313–23. doi: 10.1159/000519302 34610596

[29] Barrea L Arnone A Annunziata G Muscogiuri G Laudisio D Salzano C . Adherence to the Mediterranean diet, dietary patterns and body composition in women with polycystic ovary syndrome (PCOS). Nutrients (2019) 11(10):2278. doi: 10.3390/nu11102278 31547562 PMC6836220

[30] Vazquez ER Gomez YI Garcia E Reyes C Reyes E Camacho I . DNA Methylation in the pathogenesis of polycystic ovary syndrome. Reproduction (2019) 158:R27–40. doi: 10.1530/REP-18-0449 30959484

[31] Morford JJ Wu S Mauvais-Jarvis F . The impact of androgen actions in neurons on metabolic health and disease. Mol Cell Endocrinol (2018) 465:92–102. doi: 10.1016/j.mce.2017.09.001 28882554 PMC5835167

[32] Kim CH Younossi ZM . Nonalcoholic fatty liver disease: A manifestation of the metabolic syndrome. Cleve Clin J Med (2008) 75:721–8. doi: 10.3949/ccjm.75.10.721 18939388

[33] Xin Z Yuan JX Liu YT Long SL Mo ZC . Polycystic ovarian syndrome: Correlation between hyperandrogenism, insulin resistance and obesity. Clin Chim Acta (2020) 502:214–21. doi: 10.1016/j.cca.2019.11.003 31733195

[34] Li A Zhang L Jiang J Yang N Liu Y Cai L . Follicular hyperandrogenism and insulin resistance in polycystic ovary syndrome patients with normal circulating testosterone levels. J BioMed Res (2017) 32(3):208–14. doi: 10.7555/JBR.32.20170136 PMC6265400 29760297

[35] Torre I Bunt AE Alemán G Marquez C Diaz A Noriega L . Adiponectin synthesis and secretion by subcutaneous adipose tissue is impaired during obesity by endoplasmic reticulum stress. J Cell Biochem (2018) 119(7):5970–84. doi: 10.1002/jcb.26794 29575057

[36] Malini NA George KR . Evaluation of different ranges of LH:FSH ratios in polycystic ovarian syndrome (PCOS) - clinical based case control study. Gen Comp Endocrinol (2018) 260:51–7. doi: 10.1016/j.ygcen.2017.12.007 29273352

[37] Arkun Y Yasemi M . Dynamics and control of the ERK signaling pathway: sensitivity, bistability, and oscillations. PloS One (2018) 13(4):e0195513. doi: 10.1371/journal.pone.0195513 29630631 PMC5891012

[38] Munir I Yen HW Geller DH Torbati D Bierden RM Weitsman SR . Insulin augmentation of 17α-hydroxylase activity is mediated by phosphatidyl inositol 3-kinase but not extracellular signal-regulated kinase-1/2 in human ovarian theca cells. Endocrinology (2014) 145(1):175–83. doi: 10.1210/en.2003-0329 14512432

[39] Cadagan D Khan R Amer S . Thecal cell sensitivity to luteinizing hormone and insulin in polycystic ovarian syndrome. Reprod Biol (2016) 16(1):53–60. doi: 10.1016/j.repbio.2015.12.006 26952754

[40] Feng C Jin Z Chi X Zhang B Wang X Sun L . SHBG expression is correlated with PI3K/AKT pathway activity in a cellular model of human insulin resistance. Gynecol Endocrinol (2018) 34(7):567–73. doi: 10.1080/09513590.2017.1411474 29298529

[41] Macut D Panidis D Glisic B Spanos N Petakov M Bjekić J . Lipid and lipoprotein profile in women with polycystic ovary syndrome. Can J Physiol Pharmacol (2008) 86:199–204. doi: 10.1139/Y08-014 18418429

[42] Li H Chen Y Yan LY Qiao J . Increased expression of P450scc and CYP17 in development of endogenous hyperandrogenism in a rat model of PCOS. Endocrine (2013) 43(1):184–90. doi: 10.1007/s12020-012-9739-3 22798247

[43] Zhang X Shao H Zheng X . Amino acids at the intersection of nutrition and insulin sensitivity. Drug Discovery Today (2019) 24(4):1038–43. doi: 10.1016/j.drudis.2019.02.008 30818029

[44] Stahl EP Dhindsa DS Lee SK Sandesara PB Chalasani NP . Nonalcoholic fatty liver disease and the heart: JACC state-of-the-Art review. J Am Coll Cardiol (2019) 73(8):948–63. doi: 10.1016/j.jacc.2018.11.050 30819364

[45] Freeman AM Pennings N . Insulin resistance. In: StatPearls. Treasure Island (FL: StatPearls (2020).

[46] Diamanti KE Christakou CD Kandaraki E Economou FN . Metformin: an old medication of new fashion: evolving new molecular mechanisms and clinical implications in polycystic ovary syndrome. Eur J Endocrinol (2010) 162:193–212. doi: 10.1530/EJE-09-0733 19841045

[47] Fruzzetti F Daria P Marinella R Bucci F Gadducci A . Comparison of two insulin sensitizers, metformin and myo-inositol, in women with polycystic ovary syndrome (PCOS). Gynecol Endocrinol (2017) 33(1):39–42. doi: 10.1080/09513590.2016.1236078 27808588

[48] Kim YD Park K Lee Y Park YY Kim DK Nedumaran B . Metformin inhibits hepatic gluconeogenesis through AMP-activated protein kinase-dependent regulation of the orphan nuclear receptor SHP. Diabetes (2008) 57:306–14. doi: 10.2337/db07-0381 17909097

[49] Tao X Cai L Chen L Ge S Deng X . Effects of metformin and exenatide on insulin resistance and AMPKα-SIRT1 molecular pathway in PCOS rats. J Ovarian Res (2019) 16:12(1):86. 10.1186/s13048-019-0555-8 PMC6745801 31526389

[50] Tao X Chen L Cai L Ge S Deng X . Regulatory effects of the AMPKα-SIRT1 molecular pathway on insulin resistance in PCOS mice: an *in vitro* and *in vivo* study. Biochem Biophys Res Commun (2017) 494:615–20. doi: 10.1016/j.bbrc.2017.09.154 28988114

[51] Tao X Zhang X Ge SQ Zhang E Zhang B . Expression of SIRT1 in the ovaries of rats with polycystic ovary syndrome before and after therapeutic intervention with exenatidee. Int J Clin Exp Pathol (2015) 8(7):8276–83. PMC4555725 26339397

[52] Polak K Czyzyk A Simoncini T Meczekalski B . New markers of insulin resistance in polycystic ovary syndrome. J Endocrinol Invest (2017) 40(1):1–8. 10.1007/s40618-016-0523-8 PMC5206255 27473078

